# Advancing healthcare equity through dissemination and implementation science

**DOI:** 10.1111/1475-6773.14175

**Published:** 2023-05-23

**Authors:** Ana A. Baumann, Rachel C. Shelton, Shiriki Kumanyika, Debra Haire‐Joshu

**Affiliations:** ^1^ Division of Public Health Sciences, Department of Surgery Washington University School of Medicine St. Louis Missouri USA; ^2^ Department of Sociomedical Sciences Columbia University, Mailman School of Public Health New York New York USA; ^3^ Drexel Dornsife School of Public Health Drexel University Philadelphia Pennsylvania USA; ^4^ Brown School of Public Health and School of Medicine Washington University in St. Louis St. Louis Missouri USA

**Keywords:** dissemination and implementation science, evidence‐based intervention, health equity, healthcare equity, racism, social determinants of health

## Abstract

**Objective:**

To provide guiding principles and recommendations for how approaches from the field of dissemination and implementation (D&I) science can advance healthcare equity.

**Data Sources and Study Setting:**

This article, part of a special issue sponsored by the Agency for Healthcare Research and Quality (AHRQ), is based on an outline drafted to support proceedings of the 2022 AHRQ Health Equity Summit and further revised to reflect input from Summit attendees.

**Study Design:**

This is a narrative review of the current and potential applications of D&I approaches for understanding and advancing healthcare equity, followed by discussion and feedback with Summit attendees.

**Data Collection/Extraction Methods:**

We identified major themes in narrative and systematic reviews related to D&I science, healthcare equity, and their intersections. Based on our expertise, and supported by synthesis of published studies, we propose recommendations for how D&I science is relevant for advancing healthcare equity. We used iterative discussions internally and at the Summit to refine preliminary findings and recommendations.

**Principal Findings:**

We identified four guiding principles and three D&I science domains with strong promise for accelerating progress toward healthcare equity. We present eight recommendations and more than 60 opportunities for action by practitioners, healthcare leaders, policy makers, and researchers.

**Conclusions:**

Promising areas for D&I science to impact healthcare equity include the following: attention to equity in the development and delivery of evidence‐based interventions; the science of adaptation; de‐implementation of low‐value care; monitoring equity markers; organizational policies for healthcare equity; improving the economic evaluation of implementation; policy and dissemination research; and capacity building.

**What is known on this topic**

- Despite advances in healthcare and in dissemination and implementation (D&I) science, healthcare inequities persist.
- Many persistent inequities can be attributed to challenges and gaps in implementation across healthcare systems and contexts.
- D&I science provides rigorous approaches and methods for advancing healthcare equity.

**What this study adds**

- Four guiding principles that inform the application of D&I science to make progress towards healthcare equity.
- Eight recommendations and more than 60 associated opportunities for action that specify key directions for D&I science to partner with healthcare settings to advance healthcare equity.

## 1 INTRODUCTION

Healthcare organizations, including primary care practices, have a significant role in ensuring health equity, which is achieved when every person has the opportunity to “attain his or her full health potential,” and no one is “disadvantaged from achieving this potential because of social position or other socially determined circumstances.”^1^, ^2^ However, persistent and longstanding health inequities suggest that access to and routine provision of high‐quality healthcare is not equitably distributed across populations. According to the Agency for Healthcare Research and Quality (AHRQ) National Healthcare and Health Disparities Report,^3^ historically marginalized populations are more likely to experience care inequities (e.g., lack of health insurance coverage, higher out of pocket healthcare costs, living in areas with primary care shortages) and worse health outcomes. Additionally, some healthcare gaps are widening because of inequitable receipt of healthcare (e.g., increases in maternal mortality rate), in spite of improvements in some quality indicators (e.g., increase in percentage of people with health insurance).

To achieve health equity, we need to remove obstacles to health and well‐being for all populations across multiple sectors and societal contexts, including but not limited to healthcare settings and systems.^4^ Equity in the context of health*care*—the focus here—is defined as the “…just distribution of access to and utilization of healthcare‐related services resulting in equitable healthcare outcomes and health status among members of a population. Just distribution means without variation due to income status, race, gender, ethnicity, or other social or economic characteristics.”^5^ Healthcare inequities are shaped by broader structural factors including systemic racism^6^ and other forms of discrimination, bias, and systems of oppression (e.g., sexism, ageism, classism).^7^ These societal systems affect historical and current characteristics of health systems, healthcare delivery practices and policies, and non‐medical drivers of care that affect access and utilization. Moreover, these systems condition the underlying social determinants of health (SDOH) that include social and economic factors (e.g., housing, education, employment, neighborhood environment), which are fundamental contributors to disease and can exacerbate health inequities across these multiple sectors.^6^, ^8^ Given the factors that shape health are complex and patient‐level interventions alone are not sufficient to achieve healthcare equity,^9^, ^10^ implementation of multi‐level (e.g., individual, clinical, community), multisector (e.g., across healthcare, housing, transportation, social services), practice‐based, and policy interventions are needed to achieve an equitable healthcare system for all populations.

Evidence‐based interventions, policies, and practices (EBIs), including practice‐based evidence,^11^ are foundational to healthcare quality and outcomes. Equitable dissemination and implementation (D&I) of EBIs is a recent focus in D&I science.^12^, ^13^, ^14^, ^15^, ^16^ While referred to as D&I science,^17^ D&I are related but distinct areas. Dissemination research examines how to actively distribute information and intervention materials to a specific public health or practice audience, whereas implementation research focuses on how to accelerate the adoption and integration of EBIs into clinical settings.^17^, ^18^ To be consistent with the broader literature, we will use the D&I acronym in this paper. To conduct D&I science with a healthcare equity focus, greater attention must be given to understanding aspects of health systems that influence the availability and delivery of high‐quality care, aligned with cultural perspectives, history, assets, and needs of the community served. This requires meaningful engagement with community members, healthcare practitioners, leaders, and policy makers when developing, disseminating, and implementing EBIs.^6^, ^14^, ^15^, ^16^, ^19^, ^20^, ^21^

In 2022, AHRQ conducted a Health Equity Summit, which brought together multiple constituencies to “develop a common understanding and language to describe what health equity means in the context of health systems and what it means within the context of AHRQ's mission and healthcare delivery implementation.”^22^ This paper describes aspects of the Summit proceedings that focused on ways D&I science can facilitate equity in healthcare delivery.^23^

Prior recommendations for D&I science have included guidance for how D&I science can more explicitly focus on making progress toward health equity and calls to action to address the impact of structural racism across the broader field.^14^, ^19^, ^21^ These prior recommendations focused on a wide range of settings and approaches in D&I science, including but not limited to healthcare. Our paper builds on this prior work by addressing specific recommendations for D&I science in the healthcare setting—a critical gap in the current literature. The audience for this paper includes researchers, healthcare leaders and practitioners, and funders. We focus on two objectives that aligned with the perspectives of Summit attendees: (1) linking established equity principles that can be applied to policies and practices in healthcare systems and settings; (2) generating recommendations on the application of D&I science in advancing equity in healthcare systems and settings.

## 2 METHODS

The AHRQ Summit organizers invited a narrative review on the application of D&I science in addressing healthcare inequities as one of the key topics at the 2‐day Health Equity Summit (see Appendix A).^23^ The senior author (DHJ) invited co‐authors to create a team with complementary expertise in D&I science and healthcare equity research. A draft of the paper outline was developed through a focused literature review, guided by narrative and systematic reviews and other publications on topics relevant to this area. This generated a landscape of potential concepts and approaches for the Summit discussion. Based on our combined expertise and literature summaries, we achieved a comprehensive understanding of where foundational D&I principles and approaches would be most relevant for advancing healthcare equity and further refined these ideas based on iterative internal discussions. We also received input from Summit attendees from across the country, representing various roles in healthcare systems and academic settings (e.g., chief quality officer, physician, professor of medicine, chief health services officer). The findings from the literature review and feedback from the attendees provided the basis for generating two outcomes for inclusion in this paper: (1) guiding principles that inform the application of D&I science; and (2) recommendations for advancing healthcare equity through D&I science.

## 3 RESULTS

### 3.1 Guiding principles

**FIGURE 1:**
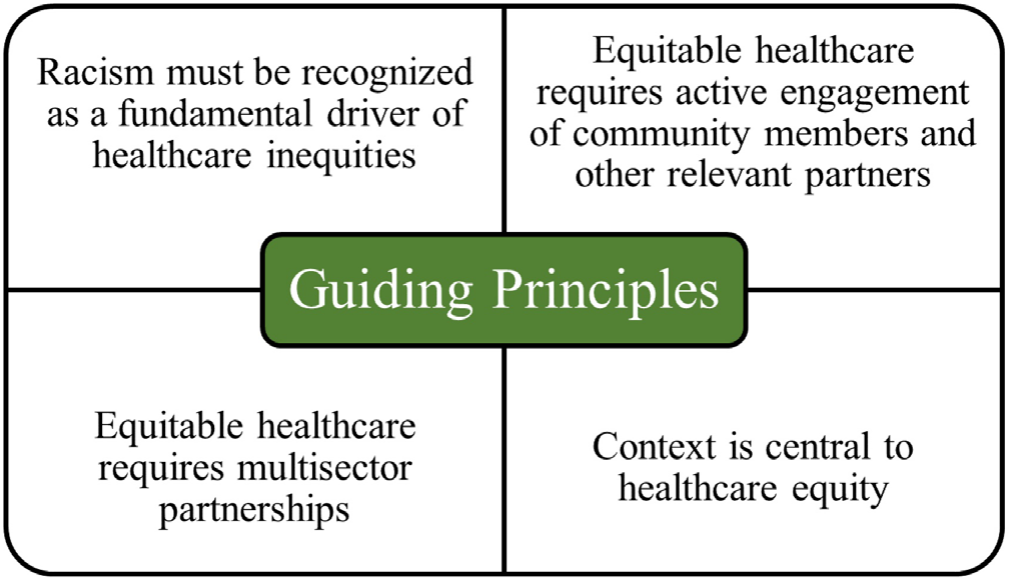
Guiding principles to achieve healthcare equity in dissemination and implementation science. [Color figure can be viewed at wileyonlinelibrary.com]

We identified four guiding principles that inform the application of D&I science to make progress towards healthcare equity. These guiding principles reflect the conceptual basis for D&I science, which is informed by multiple models and frameworks,^14^, ^19^, ^24^, ^25^ and underscore core issues raised by Summit attendees as critical to motivating healthcare equity efforts. The principles described below and in Figure 1 provide direction to equitable D&I approaches in the context of healthcare systems and settings.

#### 3.1.1 Racism must be recognized as a fundamental driver of healthcare inequities

Racism is the hierarchical structure of oppression that operates across multiple levels and sectors to create, reinforce, and maintain social and health inequities.^8^, ^26^, ^27^ To advance healthcare equity through D&I science, we must first acknowledge how racism creates and maintains inequities through institutional structures and policies that support unequal access to opportunities and societal resources.^19^ Based on this principle, we must identify ways to dismantle the effects of racism in our systems and implement more equitable policies and practices, including evidence that was informed by and reflects populations and settings impacted by racism.^8^, ^19^, ^26^, ^28^, ^29^ D&I science can provide tools for addressing systemic causes of healthcare inequities by promoting equitable and multi‐level implementation of practices and policies in healthcare systems and other relevant sectors (e.g., linking with social services to address SDOH), which have the potential to advance healthcare equity.

#### 3.1.2 Equitable healthcare requires active engagement of community members and other relevant partners

Critical to equity in healthcare research and practice is the inclusion of the voices and lived experiences of community members and patients by healthcare leaders, researchers, and providers in the development and delivery of EBIs, especially from historically underrepresented populations.^30^, ^31^, ^32^ Healthcare systems (e.g., providers and leadership) need to prioritize providing care that addresses the core barriers, social needs, and health priorities of populations that are systemically marginalized. This effort will require the ongoing engagement of key partners and constituents from across the healthcare continuum, from community members to practitioners to policy makers, to health system leaders and researchers.^21^, ^33^ Recognition of differences in power and privilege within healthcare systems will assure the value and contribution of all members and not replicate racist practices in the development and implementation of interventions.^28^, ^34^

#### 3.1.3 Equitable healthcare requires multisector partnerships

Social disadvantage affects the health of populations through a number of sectors and settings (e.g., healthcare, financial, education, and housing).^35^, ^36^ Despite huge increases in healthcare spending, health quality metrics have not improved significantly.^28^ Given the complex and interdependent social needs of populations experiencing healthcare inequities and the systems that contribute to these needs,^37^ we must focus our efforts on systemic perspectives and multisector settings that have the potential to address the underlying causes of these inequities.^38^, ^39^ Partnerships and clinical linkages with these settings can help improve some of the key barriers to both healthcare equity and health equity.^35^, ^40^, ^41^, ^42^, ^43^ For example, healthcare systems can partner with community sectors (e.g., faith‐based and/or social service organizations, tribal communities) to provide greater linkages and access to healthcare services, and to address some of the fundamental needs that must be in place to promote health. Community sectors are important for marginalized groups because they are often more trusted and may have higher reach, especially for populations that do not regularly access healthcare.^21^, ^38^, ^44^ D&I science can help enhance the delivery of EBIs through multisector settings to achieve healthcare equity.^35^

#### 3.1.4 Context is central to healthcare equity^45^, ^46^, ^47^, ^48^

Context is defined by social, organizational, political, and external factors (e.g., organizational culture, finances) that influence the successful delivery and impact of EBIs.^49^ Context includes attributes of settings in which care is delivered and the resources needed to implement EBIs equitably, such as human (e.g., staff trained), material (e.g., equipment), and economic resources (e.g., methods of reimbursement).^50^ Context is dynamic, and healthcare systems are interrelated; as such, it is important to consider and monitor the extent to which inequities may be exacerbated over time.^21^, ^51^, ^52^ D&I science can help advance understanding of the variations within systems and across settings and how they shape both inequitable implementations in healthcare and differences in health across population sub‐groups^37^, ^48^ by asking *how and in what context does this intervention work or how can the system or the intervention be adapted to work?* ^53^

These four guiding principles inform the way we conceptualize our approach to D&I science and practice with a focus on healthcare equity. These principles are embedded throughout the following recommendations.

### 3.2 Recommendations to accelerate actions for impact

**FIGURE 2:**
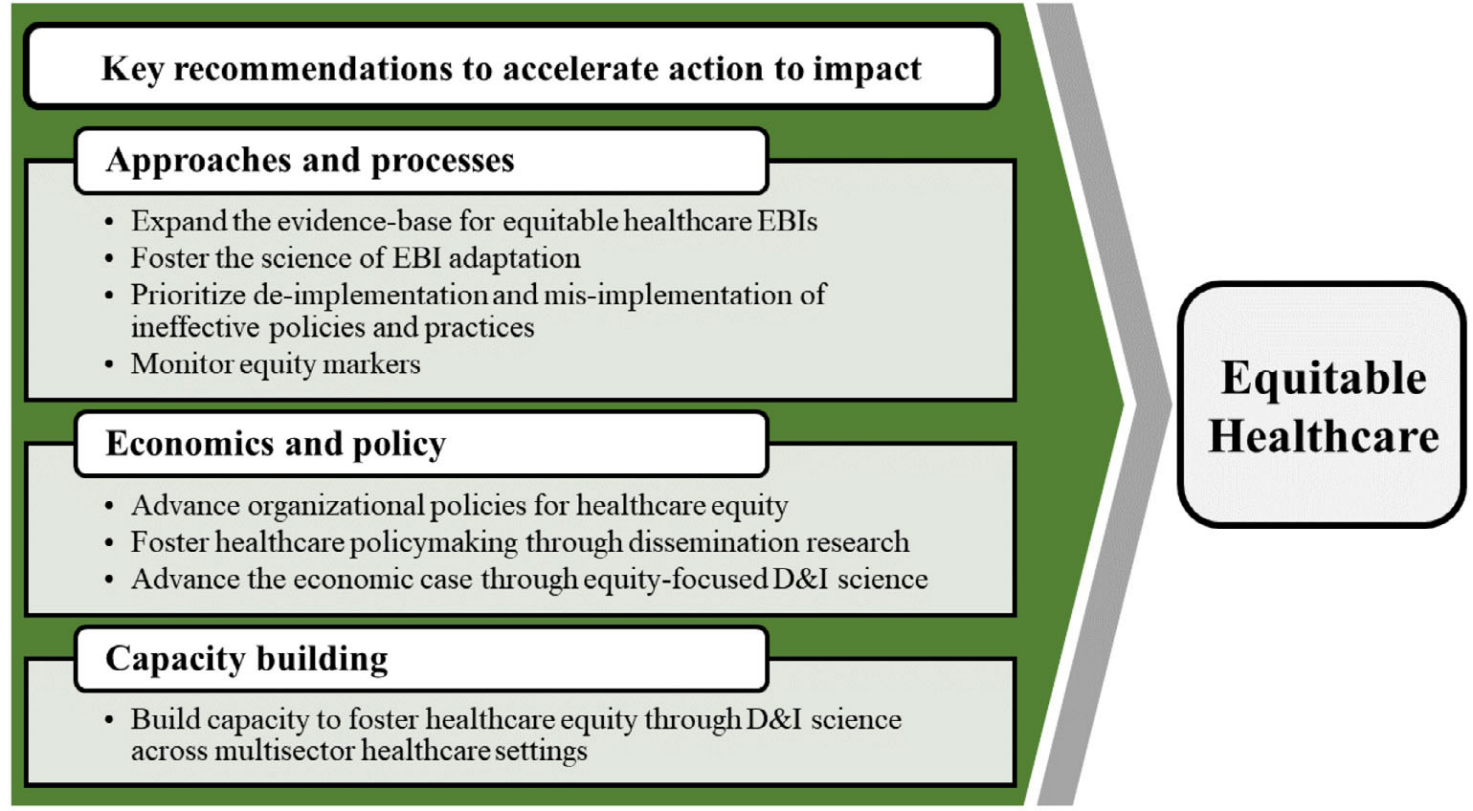
Recommendations to accelerate equitable healthcare through dissemination and implementation science. [Color figure can be viewed at wileyonlinelibrary.com]

Eight recommendations were identified of particular importance for ensuring equitable healthcare through D&I science. These recommendations, highlighted in Figure 2, fall within three domains: (1) approaches and processes; (2) economics and policy; and (3) capacity‐building. For each recommendation, we present the rationale and supporting evidence, including why it is important to healthcare equity and the role of D&I science. Tables 1, 2, 3 describe specific opportunities and examples for achieving these recommendations.

**TABLE 1:** Approaches and processes for D&I science to accelerate equitable impact.

What we know	Why it matters	Opportunities for action
**Expand the evidence base for equitable healthcare EBIs**		
Historically marginalized populations are often not included or are minimally represented in the development of EBIs.	There can be negative impacts on historically marginalized populations if they are not included in the development and testing of healthcare interventions.^54^ Healthcare interventions that work in one population (e.g., diabetes prevention program) may not work in another population and/or contribute to inequities.^55^, ^56^	Require the active inclusion of representative community members in the development of healthcare research and practice priorities.^57^, ^58^ Recruit and engage historically marginalized populations in developing, testing, and implementing EBIs.^59^ Assess how the evidence base for a specific intervention (e.g., how immunizations are delivered in a community) is determined, and the extent to which it addresses healthcare equity (e.g., preference for community care; mistrust of health systems).^60^ Implement alternative designs (e.g., mixed methods; natural experiments) and conduct rapid studies (e.g., using agile science, improvement science) and embedded research to examine the effect of systemic factors (e.g., racism) in the development of healthcare EBIs.^61^, ^62^
Interventions are developed without examining the fit with the complexity of healthcare systems.	Research protocols that do not give attention to the complexity of healthcare systems can foster disparities and are not sustainable in the long‐term.	Conduct theory‐driven evaluation of the context prior to and during the development and implementation of EBIs to improve practice. Integrate researchers in the healthcare system (conduct embedded research); decrease the silo between researchers and practitioners.^63^, ^64^
There is scant literature on the implementation of SDOH healthcare interventions—or of linkages of care—and their impact on outcomes.^65^	Without addressing the systemic causes of healthcare disparities, we will not successfully achieve equity in healthcare.	Examine the feasibility of linkages of care to implement interventions that address inequities in health (e.g., education, poverty), and structural determinants (e.g., racism).^38^, ^66^
		Use data monitoring to examine the relationship between the implementation process and the effects of SDOH (e.g., how does transportation or childcare affect the retention of patients in care).^67^ Partner with and link multiple sectors (e.g., churches and community practice networks) to increase the uptake of patients receiving healthcare (e.g., primary care visits).^15^ Examine how multisector collaborations can improve healthcare equity for marginalized populations.^68^
Technology (e.g., telehealth, eHealth, mHealth, health information) is increasingly incorporated in healthcare delivery of interventions.^69^, ^70^	As technology is increasingly being used in healthcare systems to improve care, it is important to ensure such technologies do not exacerbate inequities.^71^, ^72^, ^73^, ^74^	Leverage technology to co‐design interventions with practitioners and patients. For example, use a common and secure platform to enable providers to see lab results and diagnoses, send prescriptions, and engage in interactive communication with patients.^75^, ^76^ Use digital health (e.g., use of wearable devices, mobile health, telehealth, health information technology) to deliver patient‐centered EBI's that allow for ongoing tracking of outcomes (e.g., exercise).^75^ Research how to address barriers to digital health equity and enhance the real‐world impact of digital health tools.^71^, ^77^
Healthcare systems can partner on the development or application of EBIs in non‐healthcare settings to provide an avenue to reach marginalized communities.^41^	There are missed opportunities to increase the reach and impact of EBIs if D&I is not conducted across diverse community settings.^35^, ^41^	Test the impact of greater coordination between private sectors and nongovernmental organizations in achieving clinical and population health outcomes.^22^ Examine how issues of power, positionality of research team, organizational leadership and providers inform and affect the development and implementation of EBIs with a focus on healthcare equity.^19^, ^78^ Collaborate with marketing and health communication organizations to equitably disseminate information about EBIs.
**Foster the science of EBI adaptation**		
Implementing interventions into systems without adapting them to the context will reinforce healthcare inequities.^14^, ^21^, ^26^, ^67^	Adapting EBIs is important to ensure their fit to different contexts if we are to have equitable healthcare.^79^	Before implementing EBIs, assess the fit of the EBI with the context, especially for historically marginalized populations and settings.^14^, ^80^ When adapting EBIs and their strategies, track what is being adapted, by whom, how, when, and why it is being adapted (or not adapted).^81^, ^82^ Test different methods of tracking adaptations with a goal to reduce the burden on providers.^83^
There is limited understanding of the key components of EBIs that promote clinical effects across different settings or populations.^84^	We need to understand the impact of EBIs in different populations and the mechanisms of change across different settings.^85^ There is limited data that reports the potential negative effects of adaptations.	Develop a national database to track what is and is not being adapted, and its impact across healthcare settings.
There is limited understanding of the relationship between adaptation and fidelity of EBIs across settings.	The practical and functional aspects of measuring fidelity of EBIs are often unclear.	Be explicit about the theoretical assumptions and practical aspects of measuring fidelity in EBIs.^86^ Address cultural relevance (i.e., attention to culture, values, and interactions) when examining fidelity of EBIs for historically marginalized populations.^87^, ^88^
**Prioritize de‐implementation and mis‐implementation of ineffective healthcare**		
De‐implementation and/or mis‐implementation of healthcare interventions and policies that contribute to low‐value care is limited.^89^	Historically marginalized populations may be more likely to receive ineffective interventions.^90^	Collect evaluation data across healthcare systems and practices to examine whether interventions are implemented effectively, for whom and why. Implement guidelines calling for de‐implementation of low‐value care.^24^, ^91^ Require representation of community members from historically marginalized populations in studies of de‐implementation and mis‐implementation to inform the development of multi‐level strategies and outcome measurement.^92^, ^93^ Examine provider‐ and organizational‐level characteristics that predict the use of low‐value care^94^ and develop strategies to foster de‐implementation of low‐value care.
De‐implementation and mis‐implementation are underdeveloped areas of research and practice.^95^	There is minimal evidence to date on de‐implementation of healthcare practices and its influence on healthcare inequities.^96^	Examine the short‐, medium‐ and long‐term outcomes of de‐implementation across a variety of settings (e.g., primary care practices, federally qualified health centers), including historically marginalized communities.
**Monitor equity markers**		
Monitor equity markers related to patient social dimensions (e.g., language), demographics (e.g., gender, race) and/or setting (e.g., geography)	Most clinical systems and current standards of care do not capture the diversity of patients in their records.	Monitor who is receiving care and the type of care to ensure historically marginalized patients get the high‐quality healthcare that they deserve/need.^97^, ^98^ Evaluate how to support clinicians using technology to screen for SDOH by providing clear guidance on how to collect the data, and how to talk with patients about the data.^99^ Develop interactive dashboards that allow for visual display of SDOH and health indicators to monitor who is receiving care, and the quality of care being received. This might also include disease registries (i.e., list of patients in a given practice(s) with specific condition or procedure) to monitor equity markers (e.g., who is receiving what type of care; timely access of care, etc.).^100^, ^101^ Use improvement science methodology to support the monitoring process.^102^, ^103^ Develop dashboards to support sharing data for policy makers, healthcare, and community leaders about public health outcomes (e.g., number of infections; number of people vaccinated).^104^
Artificial intelligence (AI) has been rapidly incorporated into healthcare systems.	AI algorithms are inherently bounded by the type of data they receive; if data is biased, the algorithm will be biased.^105^, ^106^	When developing interventions, attend to bias that can happen at various phases of AI model design (e.g., labels for different groups), model training (e.g., examining whether data sets have representation of historically underserved populations), and interaction with clinicians (e.g., account for over‐reliance on AI results; account for allocation bias).^107^ Evaluate how AI interventions (e.g., diagnosis based on AI algorithms) affect patient and provider trust and relationships.^73^

Abbreviations: D&I, dissemination and implementation; EBIs, evidence‐based interventions; SDOH, social determinants of health.

**TABLE 2:** Economics and policy of healthcare equity.

What we know	Why it matters	Opportunities for action
**Advance organizational policies for healthcare equity**		
Healthcare organizations are racialized structures^108^ (i.e., organizations can reinforce structural racism through unequal distributions of resources in their macro‐, meso‐, and micro levels) and can maintain white supremacy practices.^37^	Colorblind theories (i.e., theories that do not explicitly recognize race, racism, or discrimination), permeate and foster inequities in healthcare.	Examine how current policies foster or maintain racism.^109^, ^110^ Include equity‐related constructs and measures (e.g., discrimination, racism) in assessing organizational context.^111^ Evaluate how institutional racism (e.g., excessive monitoring from supervisors, micro invalidation of peers and patients, unfairness in promotions and payment) affects the experiences of healthcare workers of color; Implement interventions and policies to address institutional racism.^112^, ^113^, ^114^, ^115^ Develop evidence to inform and improve policies that systematically examine how clinical care is being implemented for diverse patients.^116^, ^117^
Strong organizational leadership, supervision, and training focused on equity will cultivate a supportive implementation climate.^118^, ^119^, ^120^	Organizational leadership can influence healthcare policies that value equitable care and affect retention of staff,^121^ especially in under‐resourced settings.	Implement anti‐racism training for healthcare leadership and providers.^122^ Evaluate the impact of equity‐focused clinical training in leadership, supervision, and practice of a diverse workforce.^123^
**Foster healthcare policy making through dissemination research**		
Implementation is political.	The political context drives the policy making process and directly impacts healthcare equity. Laws affect access to healthcare for historically marginalized populations.	Examine how politics (e.g., reimbursement processes, immigration laws) affect access to and quality of care for historically marginalized populations.^86^, ^124^ Partner with advocacy groups and/or citizen scientists (i.e., the general public who collaborate with scientists) to understand how policies affect historically marginalized populations.^125^ Build shared measurement practices with community members, community‐based organizations, healthcare systems, service providers, and policy makers to ensure the focus on healthcare equity is relevant for the community.^126^ Examine which factors (i.e., content, tailoring materials for different audiences, who to send the materials, when materials should be sent, and how) facilitate the engagement of policy makers with research and inform policydecisions.^127^, ^128^, ^129^, ^130^
Dissemination research and policy making are important to healthcare equity.	Policy makers may not understand the perspective of those who are implementing innovations (e.g., primary care providers); dissemination research can foster understanding of the complexities of an equitable implementation process.^131^	Design and implement dissemination research with an equity‐focused approach to advance healthcare policy.^132^ Examine dissemination processes that facilitate the development and adoption of equity‐centered policies. For example, foster audience research (i.e., examine how to increase different audience's awareness, adoption, and positive attitude toward EBIs), audience segmentation research (i.e., examine how to tailor information for different audiences), and dissemination effectiveness research (i.e., examine which dissemination strategies work best for whom).^127^, ^133^, ^134^, ^135^, ^136^
Dissemination of information about EBIs will increase implementation at the provider level.	Healthcare providers may not be aware of EBIs and their variable impact among historically marginalized populations.	Engage multiple disciplines (e.g., marketing, communication, sociology, psychology) when designing EBI dissemination strategies for practitioners, community members, healthcare leaders, and policy makers.^137^, ^138^ Evaluate how different sources and formats of information (e.g., news, websites) affect providers' and organization leaders' attitudes toward healthcare equity.^135^, ^136^ Test different strategies that examine how to disseminate equity‐related information without increasing bias and racism.^123^, ^139^
**Advance the economic case through equity‐focused D&I science**		
Economic evaluation of EBIs will facilitate transparent examination of return‐on‐investment.^140^, ^141^	D&I studies should include cost data or conduct comparative economic analysis in multisector settings.^142^	Expand the use of cost‐analysis methodologies in D&I healthcare studies. Compare payment models for effect and scalability to expedite services through primary care settings (e.g., Federally Qualified Health Centers). Determine the hidden cost of services, such as parking and transportation for patients (and providers) or access to technology for different populations.^143^, ^144^
Pragmatic methods will facilitate cost analysis across multisector partners.^86^	Under‐resourced clinical and community practice organizations may not have the infrastructure to collect cost data.^142^	Use practical economic tools that will allow for comparison of implementation costs of EBIs across multiple settings of care and diverse populations.^145^ As organizations transition to value‐based payments, capture economic metrics of prevention services and evaluate how healthcare savings and expenses can impact population health.^146^
Referent perspectives (whose perspective is considered in the process) are important to calculating costs.^147^	Consideration of the needs and resource constraints of different settings can drive whether cost analyses are done and are critical to ensure healthcare equity.^148^	Develop and test rapid cycle approaches of cost estimates considering the constraints of under‐resourced settings and the potential costs of adapting EBIs.^149^ Establish learning collaboratives, with different actors (researchers, practitioners, leaders) who can play a role in bridging the quality gap between practice and research.^150^, ^151^

Abbreviations: D&I, dissemination and implementation; EBIs, evidence‐based interventions; SDOH, social determinants of health.

**TABLE 3:** Capacity building.

What we know	Why it matters	Opportunities for action
**Build capacity to foster healthcare equity through D&I science across healthcare settings**		
D&I science has fostered several capacity‐building initiatives, but most training programs are geared toward D&I researchers and are not inclusive of healthcare leaders, policy makers, practitioners or community members.^152^, ^153^	D&I training for multiple healthcare professionals, policy makers, and stakeholders is needed to foster equity in research and practice.^153^	Develop and test D&I competencies that are relevant for practitioners, healthcare leaders, community members, academic researchers, and others from the learning health system, to ensure skills and knowledge to foster healthcare equity. Develop and implement D&I trainings of different intensities (short, long), format (e.g., online, in‐person, hybrid), and focus (e.g., practitioner‐focused outcomes).
D&I trainings often do not have explicit equity domains as part of capacity building; curricula of health equity trainings do not necessarily have D&I focus.	Training across multiple groups will advance the development and uptake of equity‐focused EBIs across primary care, clinical, and community settings.^154^	Develop or adapt current D&I training mechanisms to embed explicit recognition of health equity components. Develop tools and resources to support healthcare leaders and practitioners in delivering culturally competent care among historically marginalized communities.^155^ Conduct evaluation of existing and new D&I programs to examine the impact of the training on research, capacity building, and healthcare equity.^156^, ^157^, ^158^, ^159^ Develop and test mentoring programs to engage mentors and trainees from historically underserved backgrounds in these trainings, with the goal of enhancing healthcare equity.

Abbreviations: D&I, dissemination and implementation; EBIs, evidence‐based interventions.

#### 3.2.1 Approaches and processes

##### Expand the evidence base for equitable healthcare EBIs

Often EBIs being developed and implemented in healthcare settings may not be relevant to historically marginalized populations. Historically, the evidence for EBIs is determined by randomized control trials conducted in high‐resource academic or clinical settings that do not the need of populations experiencing structural barriers to health and healthcare.^160^ These EBIs are “pushed” to healthcare systems by researchers and implemented without consideration of fit with context, feasibility, or potential for sustainability.^63^, ^137^ EBIs are also implemented without accounting for the complexity of policy and systems influences on delivery and healthcare inequities.^161^, ^162^ To achieve AHRQ's goal of ensuring access to quality, culturally‐competent, and contextually‐aligned care for historically marginalized populations,^57^ we must require increased representation of these populations in intervention development and evaluation. When developing and testing EBIs, researchers and healthcare leaders should consider designs that address the impact of context (e.g., policies and practices indicative of institutional racism) in the development and delivery of EBIs.^61^ Additionally, there is a need to conduct research in close partnership with healthcare leaders, practitioners, and patients within the settings where implementation is occurring through an embedded research model.^63^, ^163^ It is also necessary to prioritize the development and implementation of not only traditional EBIs that are researcher developed and tested, but also ‘practice‐based’ and community‐aligned EBIs.^11^, ^60^ D&I science can guide the development and implementation of interventions, including those that explicitly address inequities in healthcare systems, as well as interventions that help connect healthcare settings to critical community and social services settings.^41^, ^58^, ^164^, ^165^ Table 1 provides examples of how equity‐focused EBIs can further support AHRQ's goal of increasing efficiency, transparency, and accountability of healthcare systems.^57^

##### Foster the science of EBI adaptation

Implementing EBIs within systems without adapting them to the context, or failing to adapt the context to incorporate EBIs, will likely reinforce inequities and disparities in healthcare.^14^, ^21^, ^26^, ^67^ Adaptation is defined as thoughtful or deliberate modifications made to EBIs with the goal of improving their fit and implementation within a given context (e.g., primary care, Federally Qualified Health Centers).^81^ Adaptation is central to achieving equity as an “ongoing process of assessing needs, correcting historical inequities, and creating conditions for optimal outcomes by members of all social identity groups.”^166^ It addresses the interplay between the fit of the intervention (i.e., the what), the process of implementation (i.e., the how), and the setting in which the intervention is being implemented (i.e., the where). Adaptation is necessary to understand what the best intervention is and for whom (i.e., which populations and settings) and how EBIs produce changes in healthcare outcomes.

Adapting an EBI for a new population or setting (e.g., adding culturally appropriate content aligned with the patient's daily context or circumstances) or adapting the context to fit the EBI (e.g., integration of an intervention into the clinical workflow or electronic health record) is essential within under‐resourced healthcare settings because it takes into account the resources, infrastructure, and the broader social context that impacts inequitable implementation.^21^ A recent review of the complexities of healthcare for historically marginalized populations highlighted the adaptation of EBIs as a key determinant of the uptake of healthcare interventions.^167^ There are numerous opportunities to adapt EBIs to the needs and social contexts of historically marginalized communities.^79^, ^82^, ^83^, ^168^, ^169^ D&I science can guide when and how to adapt interventions and track adaptations across diverse settings of care. This will further inform AHRQ's goal of strengthening healthcare.^57^, ^81^ Table 1 offers additional rationale and suggested actions for advancing the science of EBI adaptation in healthcare.

##### Prioritize de‐implementation and mis‐implementation of ineffective policies and practices

De‐implementation is defined as discontinuing or abandoning practices that are not proven to be effective, are less cost‐effective than an alternative practice, or are potentially harmful.^90^ Mis‐implementation is the inappropriate continuation of programs or policies that are not evidence‐based, or the inappropriate termination of evidence‐based programs and policies.^170^ De‐implementation and mis‐implementation efforts are necessary to reduce or eliminate low‐value care, defined as “care that is unlikely to benefit the patient given the harms, cost, available alternatives, or preferences of the patient.”^171^ Low‐value care contributes to wasteful spending, which includes overuse of screening and diagnostic services and increased patient harm via over‐diagnosis, contraindicated treatments, and unnecessary financial burden.^172^, ^173^, ^174^ Patients from historically marginalized communities may be less likely to receive appropriate care and services (high‐value healthcare) and/or more likely to receive unnecessary care or services that cause financial burden.^175^, ^176^

To achieve healthcare equity, we must reverse or remove ineffective practices across healthcare settings.^177^, ^178^, ^179^, ^180^, ^181^ In doing so, we must consider the short, medium, and long‐term consequences of what is being removed or replaced from the perspective of patients. For example, de‐implementing an intervention in settings with historically limited access to quality care may exacerbate mistrust or perceptions of discrimination.^91^, ^182^ To account for these unintended consequences, particularly among systemically marginalized groups,^24^, ^183^, ^184^, ^185^ greater attention must be given to the role of culture, values, and community perspectives in de‐implementation or mis‐implementation methods and frameworks.^24^, ^185^

To accomplish AHRQ's mission of promoting the health, safety, and well‐being of people,^57^ further understanding how these factors influence the maintenance or cessation of ineffective EBIs in the context of complex healthcare settings is needed. Table 1 provides rationale and opportunities for actions to achieve this mission.

##### Monitor equity markers

To fully understand and account for the patterns and mechanisms of inequities in the healthcare system, it is important to monitor setting and patient‐level variations in health that are not attributable to clinical manifestations of disease across time and conditions.^186^ This could include tracking patterns in health outcomes and other implementation metrics related to patient social dimensions (e.g., language), demographics, and/or setting (e.g., geography).^186^ Improvement efforts should have (1) a clear, measurable marker; (2) a framework that assesses progress; (3) description of actions; (4) dedication to rapid evaluation, prediction, and learning from tests; and (5) visualization of data.^102^, ^103^ Technology can support the collection of data to summarize, standardize, monitor, and report outcome metrics, including outcomes related to the reduction or exacerbation of healthcare inequities.^75^, ^186^ For example, electronic health records can be leveraged to provide metrics around SDOH to inform patient‐centered care,^100^ and natural language processing has been used to capture SDOH markers from clinical notes.^187^ However, when developing monitoring platforms, attention is needed to not replicate bias and foster disparities in care.^105^, ^188^ Additionally, D&I science can support the development of monitoring processes by assessing different perspectives (e.g., patient, provider) on the acceptability and feasibility of screening and monitoring of SDOH.^189^ Monitoring equity markers can help achieve AHRQ's goals of increasing high quality of care, and expanding equitable access to comprehensive, community‐based healthcare services.^22^ Table 1 offers additional rationale and suggested actions for monitoring equity markers in healthcare.

#### 3.2.2 Economics and policy

##### Advance organizational policies for healthcare equity

Implementation of EBIs is shaped by national, state, local, and healthcare system policies.^61^ These policies can influence the misalignment of organizational incentives, national regulations around healthcare metrics, or competing priorities among providers.^190^, ^191^ Additionally, organizational leadership,^118^, ^192^, ^193^ capacity,^194^, ^195^, ^196^ and culture and climate^197^, ^198^, ^199^ within healthcare settings affect the uptake, implementation, and sustainment of EBIs. How different organizations collaborate (or compete) in the delivery of services also affects equitable policies and service delivery.^200^, ^201^ Importantly, forms of structural and institutional racism also shape the policies and practices of healthcare organizations.^19^ Systemic racism contributes to the accumulation of resources and power in healthcare structures that promote healthcare inequities and that are reinforced by following existing norms, practices, laws, and bureaucratic structures, (meaning) healthcare systems can perpetuate racism.^108^, ^109^, ^202^, ^203^, ^204^

D&I science can enhance our understanding of the role of policies and policy making in the implementation of EBIs, including how issues of power, mistrust, and bias that operate through institutional practices/policies shape inequitable healthcare delivery.^34^, ^205^ For example, D&I science can provide evidence for policy change and guide requirements that demonstrate measurable actions toward the elimination of racial inequities in healthcare organizations (e.g., instituting mandatory trainings on cultural competency and anti‐racism, establishing metrics for diversity in the healthcare workforce, and establishing accountability processes).^206^ D&I science can also support the implementation of requirements that assure program integrity and equity, one of the goals of AHRQ.^22^ Table 2 offers additional rationale and suggested actions for advancing organizational policies for healthcare equity.

##### Foster healthcare policy making through dissemination research

The uptake of EBIs is informed and impacted by the political context which drives persistent healthcare inequities.^131^, ^207^, ^208^ Politics shape a wide range of healthcare outcomes including access to healthcare through appropriations, regulation, administration, subsidization, and allocation of resources.^207^ To strengthen the healthcare system,^57^ we need to understand and promote policies that affect equitable healthcare. Dissemination research (i.e., examining how to actively share information about EBIs with a specific population) can help shape policy.^133^, ^134^, ^209^ However, research on how to effectively disseminate information about the equitable implementation of EBIs in healthcare is yet to be fully examined.^127^ Research is needed to evaluate the influence of policies (e.g., primary care reimbursement processes) on the access and quality of healthcare for historically marginalized populations.^61^, ^210^, ^211^, ^212^, ^213^, ^214^

Dissemination research can help increase awareness, knowledge, and motivation of policy makers to translate research into action^61^ and create positive conditions (e.g., resources, governance) for the adoption of equity‐focused initiatives.^214^, ^215^ However, policy makers often have different levels of understanding about healthcare, EBIs, values, and priorities regarding health equity.^214^, ^216^ Additionally, low levels of knowledge about health inequities and limited beliefs about their existence^217^, ^218^ can affect provider care^123^ and policies that foster equitable solutions.^214^ Dissemination research can advance understanding of how to reach and promote best practices that increase uptake of evidence‐based information by practitioners and policy makers and inform policy actions (e.g., payment reforms that will improve healthcare value and equity).^128^, ^131^, ^216^, ^219^, ^220^ Table 2 provides additional examples of actions that can be taken to foster healthcare policy making.

##### Advance the economic case through equity‐focused D&I science

As Proctor and colleagues^221^ state: “someone must pay if any new discovery or improvement to care is to be adopted, sustained, and scaled.” Cost is a key aspect to consider in developing and implementing interventions.^222^ Economic considerations are important because healthcare leaders, practitioners, and policy makers may be hesitant to invest in implementing EBIs without knowing the return‐on‐investment and/or their perceived value. Despite this, relatively few studies include cost data or routinely conduct comparative economic analysis.^142^

D&I science, and the fields of business and economics, emphasize the importance of financial resources in the development and implementation of EBIs across healthcare settings.^209^, ^223^, ^224^ Explicit consideration about the “referent,” or whose perspective is most important when calculating costs.^108^, ^147^, ^225^, ^226^ Who decides what to pay for, when, and how, are questions that highlight the economic infrastructure that drives the implementation of EBIs. Consideration of cost is critically important to equitable implementation, especially in low‐resource settings (e.g., federally qualified health centers) with significant budget constraints.^147^ Systematic methods of estimating costs of EBI implementation can enable planning and budgeting in healthcare systems and organizations^227^, ^228^, ^229^ while balancing speed, efficiency, and rigor based on those who need the information the most.^54^, ^150^ Cost estimates need to assess core elements of EBIs including contextual constraints and EBI adaptation,^150^, ^230^ as well as the implementation of innovations in care (e.g., telehealth).^149^ Tools have been developed to support capturing such cost data; however, practical approaches to collect cost data across under‐resourced healthcare settings are lacking.^142^, ^231^ Table 2 highlights opportunities for action related to D&I, economics, and healthcare equity.

#### 3.2.3 Capacity building

##### Build capacity to foster healthcare equity through D&I science across multisector healthcare settings

Primary care practitioners, healthcare leaders, and researchers have been at the forefront of the changes in innovations in healthcare delivery^86^ (e.g., patient‐centered care,^232^ the use of electronic medical records,^233^ payment redesign^234^). Advancing the use of D&I science by these groups will promote equity in healthcare practice and research. However, to achieve this goal, there needs to be a workforce able to generate and apply knowledge that is responsive to community needs, accelerates the implementation of EBIs, and promotes the delivery and monitoring of equitable healthcare.^235^

It is important for researchers to be embedded within and knowledgeable of the healthcare system to collaborate and co‐create insights and evidence to improve healthcare delivery.^236^ AHRQ has recommended a series of competencies for healthcare scientists,^236^ which include knowledge and skills about D&I science. Several capacity‐building initiatives, from graduate courses to 2‐year training programs, have been developed for D&I researchers.^152^, ^153^, ^156^, ^237^, ^238^, ^239^ Additionally, more recent reviews on D&I capacity building have underscored the need for training practitioners, policy makers, and others across a variety of educational levels.^153^, ^240^ However, D&I training needs from the perspectives of these stakeholders (e.g., practitioner, researcher, administrator, policy maker) are unknown.^241^

Central to advancing healthcare equity is an effectively‐trained workforce with the skills to ensure equitable implementation and monitoring of EBIs.^22^ Despite the attention to equity in D&I science, opportunities for training in equity‐focused D&I science are few.^242^ Different formats (e.g., workshops, semester classes, two‐year trainings), types of trainings (e.g., online, in‐person), and their relevance to the needs of target audiences (e.g., providers, community members, policy makers) will build capacity and assure opportunity for healthcare equity across different settings.^21^, ^240^, ^243^ Healthcare leaders can partner with academia to develop training with the goal of promoting the development, synthesis, and adoption of EBIs into practice^154^ and that is practice‐based. Rigorous training, with an explicit focus on healthcare equity and D&I science (among other fields), needs to be further developed, tested, and made available to ensure equitable implementation of healthcare.^244^ Table 3 highlights opportunities to build capacity in D&I science across healthcare settings.

## 4 DISCUSSION

The persistent and longstanding health inequities affecting marginalized groups in the United States reflect a pervasive problem of how healthcare is implemented. There is an urgent need for a seismic shift in the current approaches to understand and correct healthcare inequities.^245^ This paper is one outcome of the AHRQ Health Equity Summit; it draws upon scientific literature and expert input to operationalize the application of D&I science to achieve healthcare equity. This process was informed by four guiding principles considered central to the role of D&I science in advancing healthcare equity. These principles are embedded throughout eight recommendations to expand the role of D&I science in healthcare equity, which fall within three domains: (1) approaches and processes; (2) economics and policy; and (3) capacity building.

We acknowledge the limitations of this paper, as the review is restricted in its scope and did not allow for a comprehensive exploration of themes or an in‐depth analysis of each of the principles and recommendations presented. It also did not permit discussion of all the elements and broader societal systems that can lead to healthcare inequities and could be addressed through a focus on D&I science and healthcare equity. However, the approach and findings are well grounded in expert publications on the relevant topics and further enhanced by direct consultation with the expert attendees at the AHRQ Summit.

## 5 CONCLUSION

Despite advancements, problems with healthcare interventions contribute to persistent healthcare inequities. D&I science offers a more immediate pathway to action, which will advance healthcare equity.^246^, ^247^, ^248^ We propose that every healthcare practice, program, or policy should have an equity focus or considerations, and D&I science can ensure equitable implementation of EBIs. The opportunities for action highlighted across the D&I domains provide a path forward to ensure more rapid advances in healthcare equity.

## Supporting information


**Appendix A.** OVERVIEW PAPER *HSR* SUPPLEMENT (AGENCY FOR HEALTH CARE RESEARCH AND QUALITY EQUITY)
